# Willow phenological modelling at different altitudes in central Italy

**DOI:** 10.1007/s10661-020-08702-7

**Published:** 2020-10-30

**Authors:** Fabio Orlandi, Luigia Ruga, Marco Fornaciari

**Affiliations:** Department of Civil and Environmental Engineering, University of Perugia, Borgo XX Giugno 74, 06121 Perugia, Italy

**Keywords:** Phenological modelling, *Salix* species, leaf period, Meteorological variables

## Abstract

In order to estimate the impact of climate change on the phenological parameters and to compare them with the historical record, a decision support system (DSS) has been applied employing a Phenological Modelling Platform. Biological observations of two willow species (*Salix acutifolia* and *smithiana* Willd) in 3 gardens at different altitudes located in Central Italy were utilized to identify suitable phenological models related to four main vegetative phase timings (BBCH11, BBCH91, BBCH 94, BBCH95), and male full flowering (BBCH 65) clearly identifiable in these species. The present investigation identifies the best phenological models for the main phenophases allowing their practical application as real-time monitoring and plant development prediction tools. Sigmoid model revealed high performances in simulating spring vegetative phases, BBCH11 (First leaves unfolded), and BBCH91 (Shoot and foliage growth completed). *Salix acutifolia* Willd. development appeared to be more related to temperature amount interpreted by phenological models in comparison to *Salix smithiana* Willd. above all during spring (BBCH11 and 91), probably due to a different grade of phenotypic plasticity between the 2 considered species.

## Introduction

Plant phenological development is mainly influenced by meteorological and climatic factors (Keller 2015), and temperature, solar radiation and precipitation may have a great influence on vegetative and reproductive development, yields and fruit characteristics (Kozlowski and Pallardy 1997; Moriondo et al. 2015; Orlandi et al. 2020). Temperature represents the main factor in the phenological development of plants, which allows the creation of efficient interpretative models for a wide range of species in many different countries and regions (Chuine et al. 2013). Various plant species developments are modelled to predict phenophases and to assess the impact of temperature change on plants (García de Cortázar-Atauri et al. 2010; Ibañez et al. 2010; Pearson 2019; Rojo et al. 2020).

In these studies, the availability of standardized data is essential to assure the replicability of the same monitoring. The “International Phenological Gardens” network through protocols adoption furnish detailed professional observation-guide (http://ipg.hu-berlin.de) and the methodology for following the phenological observations is the same in all the gardens and the references toward the international standardized BBCH method is really recommended. Specific sections of the Phenological Gardens are dedicated to clone plants derived by vegetative propagation through stem cutting of trees and shrubs common to all the network gardens, in order to record only the environmental influence on their development. The first arboreal species utilized in Italian gardens were willows (*Salix acutifolia* Willd*.*, *Salix smithiana* Willd*.* and *Salix viminalis* L.) which came from central Europe. There is a progressive increase in willow diversity from south to north with the median number of taxa per stand in southern Europe being three, and six in northern Europe (Cronk et al. 2015).

Several models have been used during the last 60 years to simulate phenological development stages; for flowering and fruit setting, the conventional linear growing degree-days model (GDD) was initially proposed by Amerine and Winkler (1944). This simple temperature accumulation model uses daily mean temperatures, with a fixed base temperature and onsets temperature accumulation at fixed dates. Some simulations based on the Winkler model and the Wang and Engel curvilinear model (García de Cortázar-Atauri et al. 2010) were formulated and tested to interpret the flowering phenomenon.

In particular, willows show a strong thermal control over flower buds very sensitive to the first heat amounts during the winter ending period, so GDD accumulations can be very useful to predict reproductive phenology, and the annual flowering cycle reliance on temperature amounts demonstrates the genetic control over phenological response (Mosseler and Papadopol 1989).

Phenological models may also be paired to climate change scenarios so as to project their potential impacts on phenological timings (Ziello et al. 2012; Fu et al. 2015). Therefore, it is critical to understand to what extent temperature may influence the timings of the reproductive and vegetative cycles, as well as identifying plant varieties that better adapt to the climatic changes. The purpose of this study was to determine and analyse the phenological growth stages of two willow species (*Salix acutifolia* and *smithiana* Willd.) in comparison to the climate characteristics recorded in three Phenological Gardens of central Italy included in the International Phenological Gardens network (IPG). The phenological records from 2005 to 2018 were used to identify suitable phenological models related to four mains vegetative phenological timings (in spring and fall) and flowering of the two willow species grown in the phenological gardens.

## Materials and methods

### Study area and datasets

**Fig. 1:**
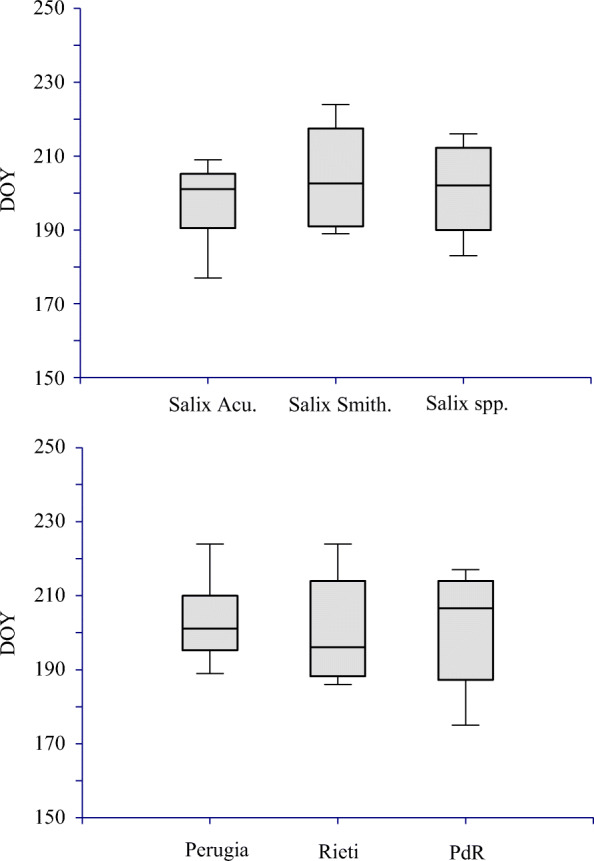
Box plots of the leaf periods (LP) for Salix acutifolia, smithiana and their mean values, calculated as difference between BBCH 95 and BBCH 11. Inter-species and inter-site (Perugia, Rieti, Pian di Rosce) phenological data

Phenological observations from three phenological gardens in central Italy were used for model calibration by monitoring indicator plants left to grow in a Inatural way for as long as possible (Orlandi et al. 2014) (Fig. 1). The first phenological garden, located 15 km from Perugia (Umbria Region, central Italy) in an area characterized by Mediterranean climate, is one of the oldest Italian ones and includes local, national and some indicator species common to all IPGs (Schnelle and Volkert 1964) planted since 1994. The garden has the following geographical coordinates: latitude, 43° 00′ 40″ N; longitude, 12° 14′ 52″ E, and its total area is of about 4000 m^2^. The second phenological garden considered is that located near Rieti (Lazio Region, central Italy, lat: N 42° 25′ 30″; Long: E 12° 49′ 45″; Alt. about 270 m a.s.l.) with an area of about 2000 m^2^. In this second garden the planting of the vegetative species is more recent (2005). The third garden, of about 3000 m^2^, is named Pian di Rosce (PdR) and its the more elevated, being located in the Terminillo Mountain (Lat: N 42° 28′ 40″; Long: E 12° 56′ 28″) 15 km from Rieti at an elevation of about 1050 m a.s.l. Here some guide species were planted since the early 2000s.

The 2 willow species present in all the phenological gardens and considered by the study were as follows:

- *Salix acutifolia* Willd*.* Family: Salicaceae; common name: violet willow, sharp-leaf willow; a deciduous shrub or a small tree up to 10 m high flowering during February–April, before the bud burst, and fructifying in May–June. According to what described by Rahmonov (2016) S*alix acutifolia* seems to be a species that prefers sandy areas typical of humid environments and appears to be a primary colonizing species in its distribution range.
- *Salix smithiana* Willd. Family: Salicaceae; common name: Smith’s willow; a deciduous shrub or a small tree up to 9 m high flowering during February–April, before the bud burst, and fructifying in May–June.

The *Salix smithiana* species is a hybrid species that derives from the crossing of different species of willow such as Salix caprea × viminalis, *Salix smithiana* for this reason can also be found in our Mediterranean habitats as seen in GBIF Secretariat (https://www.gbif.org).

The plant species were derived several years ago from mother plants received from the German Weather Service as European coordinator for the phenological gardens activities. For model calibration (parameter fitting), temperature records from the meteorological stations installed near the different gardens and over the period of 14 years (2005–2018) were considered. The daily meteorological data for the Perugia garden were provided by the station of the Italian Agrometeorological Network (datalogger DA 9000) located about 50 m from the phenological garden. The data for the two Phenological gardens of Rieti and Terminillo Mt. were supplied directly by the meteorological stations located nearby the gardens themselves and managed by the “Apennines Centre of Terminillo Mt.” of the University of Perugia (http://www.cat.unipg.it). Furthermore, in order to enhance the statistical robustness of the selected models for a given willow species, the phenological timings for the same species for all three sites were considered for model calibration.

### Phenological phases and models

Every biological phase was interpreted by a phenological point of view through observations realized directly in the phenological gardens utilizing the international conventional “BBCH” key to obtain comparable values as reported in literature (Chmielewski and Roetzer 2001; Meier 2001; Saska and Kuzovkina 2010). In the present study, the following phenological phases were considered for the vegetative cycle of both *Salix* species: BBCH11, first leaves unfolded; BBCH91, shoot growth completed; foliage still fully green; BBCH 94, 50% of leaves discoloured; BBCH95, leaves have mostly fallen (50% of leaves have fallen). Also, BBCH 65 (male full flowering) was reported to highlight reproductive developments during the study period. In each garden, observations were conducted on three individuals of each *Salix* species, to limit random variability, and the mean date of the onset of each phenophase was calculated as an average of the three plants. Moreover, two principal Leaf presence Periods (LP) were calculated as the differences between the BBCH95-11 dates and BBCH95-91 dates during the study years in the 3 areas. In particular, LP represented the existence of efficient leaves by a physiological point of view considering that phase BBCH 95 is referred to 50% of leaves fallen with the other in a very advanced senescence phase.

An evaluation of the efficacy of the interpretation by the statistical models to follow the biological trends during the yearly LP development (with a step of 5% of the total LP) was realized considering both *Salix* species in the 3 gardens during the whole study period. The aim of this evaluation was to identify averagely the potential presence of a better simulated LP stage during the entire vegetative season.

Various models were used to simulate thermal accumulation during the considered phenological phases, and the freely adjusted best-fit parameters for different phenological forcing models (GDD, Richardson, Sigmoid and Wang) were selected considering that their performances can be significantly more efficient than those of the default parameters (Caffarra et al. 2011). The best performing models for each phenological phase were selected.

### Phenological trend analysis

A trend analysis of the two willows phenological series was realized using nonparametric Mann-Kendall tests, for monotonic positive or negative trends. Positive *Z* values reveal the presence of a delay trend in the phenological data, while negative *Z* values indicate an advance trend for the same data. The estimation of a true slope of an existing trend (as change per year) was realized through the Sen nonparametric method. The significance levels were represented by the following symbols: *, trend at *p* < 0.05; **, trend at *p* < 0.01; and ***, trend at *p* < 0.001. The Mann-Kendall trend analysis was realized with the Excel template application MAKESENS version 1.0 (Salmi et al. 2002).

### Modelling tools and performance verification

In this study, the Phenological Modelling Platform, PMP (Garcia de Cortazar-Atauri et al. 2010; Chuine et al. 2013; Costa et al. 2019), was used to test and calibrate different phenological models applied to both *Salix* species. PMP was considered and employed as a useful interactive software-based system (decision support systems, DSS) for decision-making with the use of large volumes of environmental information. The platform allowed to apply and test different phenological models using climatic and phenological data for calibrating the same models and estimating the best-fit model parameters in the specific investigation area. PMP evaluates best-fit parameters on the base of the simulated annealing algorithm of Metropolis et al. (1953) through an iterative optimization procedure.

For each specific location, the environmental and biological data used were daily mean (Td), maximum (Tmax) and minimum (Tmin) temperatures, latitude and observed phenological timings for leaves growth and their senescence and flowering (in days of the year, DOY). The model performance is expressed by the root-mean-square error (RMSE), Nash-Sutcliffe coefficient of efficiency (EF) and then the Akaike Information Criterion (AIC). Particularly, Nash-Sutcliffe efficiency is a normalized statistic that determines the relative magnitude of the residual variance (“noise”) compared with the measured data variance (“information”) showing the effectiveness of the simulated data reconstruction (Nash and Sutcliffe 1970; Moriasi et al. 2007; Costa et al. 2019). EF varies between – ∞ and + 1, + 1 corresponds to an ideal fit, 0 corresponds to the performance of the null model (average), while a negative value to a worse prediction than the null model.

## Results

### Phenological model performance

The best models for each corresponding vegetative and reproductive phenophases are shown in Table 1 considering at the same time both the *Salix* species data and the 3 garden areas from 2005 to 2018. EF values evidenced the sigmoid function as the more efficient in the interpretation of all the phenomena in comparison to other models (Caffarra et al. 2011). The *T*_0_ parameters (starting dates of the model calculation for each phase) were always converted to a photoperiod evaluation allowing starting dates to vary with the latitude of the sites. PMP calculated a photoperiod using the latitude value of each site during the calculation period, considering whether *T*_0_ was situated during the increasing day length period (DLP) for spring phenological phases (BBCH 11, 91, 65) or during the decreasing DLP (BBCH 95) (Caffarra et al. 2011).

**Table 1:** Best phenological models for the principal phases (BBCH11, 91, 95, 65) considering both *Salix acutifolia* and *Smithiana* in the 3 phenological gardens

	Rep	t0	Sstar	Function	Parameters			SStot	SSres	RMSE	EF	NbObs
BBCH 11					d	e	------					
	18	11.148	25.000	Sigmoid	− 40.000	5.690	------	14306	6143	8.55	0.571	84
					Tb	------	------					
	1	11.195	248.744	GDD	0.729	------	------	14306	6315	8.67	0.559	84
					Tlow	Thigh	------					
	11	11.203	248.654	Richardson	0.732	22.333	------	14306	6315	8.67	0.559	84
					Topt	Tmin	Tmax					
	1	9.428	29.328	Wang	29.803	− 39.489	44.012	14306	6268	8.64	0.562	84
BBCH 91					d	e	------					
	5	11.156	92.553	Sigmoid	− 0.855	4.284	------	7019	2452	5.40	0.651	84
					Tb	------	------					
	1	14.793	141.350	GDD	8.336	------	------	7019	3065	6.04	0.563	84
					Tlow	Thigh	------					
	9	14.789	142.284	Richardson	8.296	20.862	------	7019	3041	6.02	0.567	84
					Topt	Tmin	Tmax					
	1	9.492	115.138	Wang	22.005	− 40.000	43.517	7019	2471	5.42	0.648	84
BBCH 95					d	e	------					
	7	13.845	26.909	Sigmoid	− 0.049	29.876	------	21656	16197	13.89	0.252	84
					Tb	------	------					
	6	11.718	423.700	GDD	0.000	------	------	21533	17349	14.37	0.194	84
					Tlow	Thigh	------					
	1	11.803	437.239	Richardson	0.003	25.702	------	21533	17333	14.36	0.195	84
					Topt	Tmin	Tmax					
	5	13.804	59.205	Wang	30.000	− 40.000	48.516	21533	15996	13.80	0.257	84
BBCH 65					d	e	------					
	9	8.786	70.903	Sigmoid	− 0.093	− 21.537	------	7390	6682	8.92	0.096	84
					Tb	------	------					
	10	11.963	7.371	GDD	1.115	------	------	7390	7328	9.34	0.008	84
					Tlow	Thigh	------					
	13	11.963	9.068	Richardson	0.018	23.074	------	7390	7321	9.34	0.009	84
					Topt	Tmin	Tmax					
	10	8.943	78.058	Wang	10.580	− 39.803	40.426	7390	6588	8.86	0.109	84

Spring vegetative phases (BBCH11 and BBCH91) were those with the best EF values, while the senescence phase (BBCH95) and the reproductive phase (BBCH65) showed lowest values. In particular, full flowering (BBCH65) showed very low values with all the utilized models highlighting how difficult the interpretation of the reproductive process is with the only use of temperature values.

In Table 2, the interpretation of the principal phenophases was realized separately for *Salix acutifolia* and *Salix smithiana* utilizing only sigmoid function. Moreover, at first the modelling was realized using only the temperature variable and then considering the Day Length variable; in this case, the Bidabe function was utilized for the second variable. *Salix acutifolia* showed better results than *Salix smithiana* for all the phenophases with EF values of 0.79 for BBCH11, 0.75 for BBCH91 and 0.55 for BBCH 95. The full flowering phase did not show sufficient results, as well in neither of the 2 species.

**Table 2.** Sigmoid models also with Day Length variable for the principal phenophases (BBCH11, 91, 95, 65) considering *Salix acutifolia* and *Smithiana* together and singularly.

		Rep	t0	Sstar	Phase function (1)	d	e	Phase function (2)	d (sig) q10 (bid)	SStot	SSres	RMSE	EF	N Obs
Salix acutifolia (Temp)	BBCH 11	2	8.88	12.06	Sigmoid, Tmean	− 0.10	25.08	------	------	11396	2450	7.64	0.79	42
	BBCH 91	3	11.13	89.01		− 40.00	5.63	------	------	4953	1249	5.45	0.75	42
	BBCH 95	2	15.11	105.69		− 0.17	5.75	------	------	7180	3266	8.82	0.55	42
	BBCH 65	7	11.38	14.00		− 40.00	4.03	------	------	2302	1716	6.39	0.25	42
S. acutifolia (Temp; Day Length)	BBCH 11	1	8.88	344.87	Sigmoid, Tmean	− 40.00	5.65	Bidabe, Day Length	0.54	11396	2374	7.52	0.79	42
	BBCH 91	15	8.07	471.41		− 40.00	5.65		0.86	4953	1140	5.21	0.77	42
	BBCH 95	14	15.11	128.39		− 36.86	13.60		4.94	7180	2417	7.59	0.66	42
	BBCH 65	16	10.85	44.21		− 40.00	5.65		− 23.87	2302	1623	6.22	0.29	42
Salix smithiana (Temp)	BBCH 11	18	11.13	29.70	Sigmoid, Tmean	− 31.51	5.79	------	------	2907	1433	5.84	0.51	42
	BBCH 91	1	8.89	141.70		− 0.09	− 12.53	------	------	1952	724	4.15	0.63	42
	BBCH 95	8	15.18	54.35		− 0.03	27.49	------	------	13048	5686	11.64	0.56	42
	BBCH 65	8	8.94	78.29		− 40.00	− 7.50	------	------	4332	3399	9.00	0.22	42
S. smithiana (Temp; Day Length)	BBCH 11	6	9.24	197.85	Sigmoid, Tmean	− 38.36	6.38	Bidabe, Day Length	1.08	2907	1436	5.85	0.51	42
	BBCH 91	1	10.40	338.80		− 40.00	6.20		0.97	1952	666	3.98	0.66	42
	BBCH 95	11	15.17	174.74		− 40.00	22.36		1.37	13048	5123	11.04	0.61	42
	BBCH 65	13	8.97	76.05		− 40.00	− 7.75		− 10.89	4332	3411	9.01	0.21	42

The addition of Day Length in the model functions caused only a small increase in EF values apart from the autumnal senescence phase (BBCH95) which moved from 0.55 to 0.66 for *Salix acutifolia* and from 0.56 to 0.61 for *S.smithiana*. Moreover, the model performances are generally higher for *acutifolia* than for *smithiana*, which may suggest that the latter is less sensitive to thermal forcing.

In figure 1 the leaf periods (LP) box plots shown have been calculated year by year, as the difference between BBCH95 and BBCH11 dates. The box plots refer to the singular species mean values in the 3 gardens (inter-species phenological effect). The LP median values were around 200 days, considering all the species, while the range of distribution of these data was of 20 days, in average (190–210 days). Moreover, inter-site (Perugia, Rieti, Pian di Rosce) phenological effects are shown considering both of the species.

In addition, different LP percentages (with a step of 5% of the total LP) were calculated year by year considering both *Salix* species in the 3 gardens during the whole study period. The yearly dates (DOY) related to the different LP stages were utilized in the simulation by the modelling platform.

**Fig. 2:**
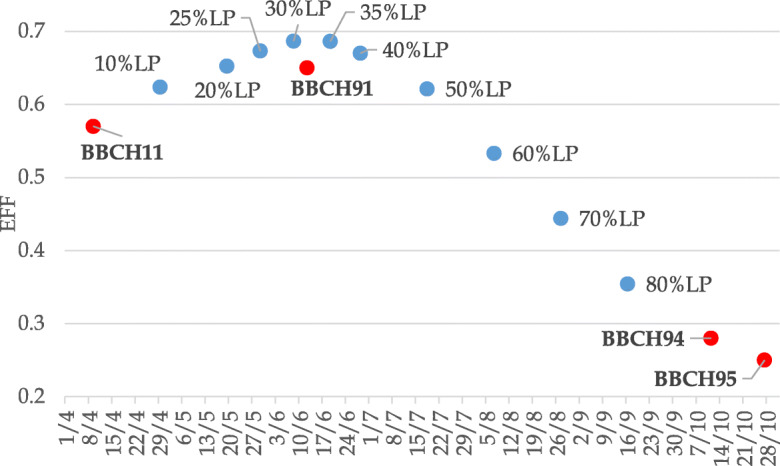
Efficiency values of the vegetative phases (BBCH 11, 91, 94, 95) and of the progressive percentages of leaf period (LP) as mean of the two Salix species during the entire study period in the 3 gardens

The sigmoid function of the utilized model expressed EF values of the vegetative phases (BBCH11-91-94-95) and of progressive LP percentages that were represented in a unique chart (Fig. 2) consequently during the year. Averagely the BBCH91 date (adult leaves phase) resulted included between the 30–35% of the LP total period when the progressive percentages of leaf period reached the highest EF values manifesting the best interpretation efficacy of the models to follow the biological trends during this period of the year.

### Phenological trend results

**Fig. 3:**
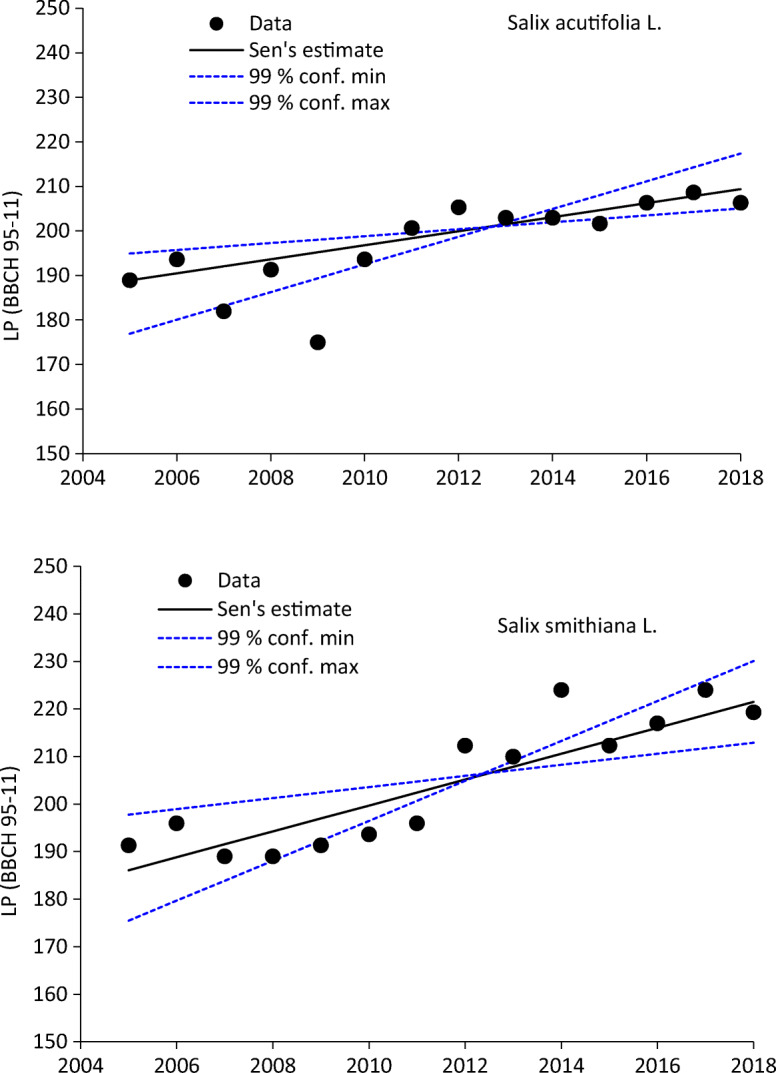
Trend analysis of both Salix species leaf period interval (BBCH95-11)

The trend analysis results concerning all the BBCH phases and the Leaf presence Periods (BBCH95-11; BBCH95-91) for each *Salix* species in the 3 gardens are shown in Table 3. Vegetative phases (BBCH11-91-95) showed significant trends (Table 3) also confirmed by variable LP. Even if the DOYs related to the different phases in each garden were clearly different considering their various altitudes, their historical series presented very similar trend values, justifying a common interpretation. BBCH65 trend was significant only for *Salix smithiana*. Spring phases showed a negative trend (BBCH11-91-65) due to a progressive advance of their annual dates, while autumnal phase (BBCH95) showed a positive trend determined by a progressive delay of senescence phenomena (leaves colouring and fall). Leaves presence periods were progressively longer, determining significant positive trend values, graphically represented by Fig. 3, as well.

**Table 3.** Trend analysis (Mann-Kendall test) of the BBCH phases (included LP).

Mann-Kendall trend				
	Time series	Test *Z*	Signific.	*Q*
Salix Acutifolia	BBCH11	− 3227	**	− 1167
	BBCH91	− 3392	***	− 0875
	BBCH94	0785		0259
	BBCH95	1781	+	0515
	BBCH60	− 0223		0000
	BBCH65	0057		0000
	LP (BBCH95-11)	3404	***	1576
	BBCH95-91	3015	**	1152
	BBCH91-11	2433	*	0389
Salix Smithiana	BBCH11	− 3461	***	− 1167
	BBCH91	− 2878	**	− 0583
	BBCH94	1770	+	0778
	BBCH95	2776	**	1458
	BBCH60	0995		0583
	BBCH65	2011	*	0778
	LP (BBCH95-11)	3525	***	2722
	BBCH95-91	3624	***	2333
	BBCH91-11	3017	**	0778

As far as it concerns BBCH11 evidence derived by the Platform, the sigmoid model provided values that were highly conditioned by the strong negative trend manifested during the 14-year study period.

**Fig. 4:**
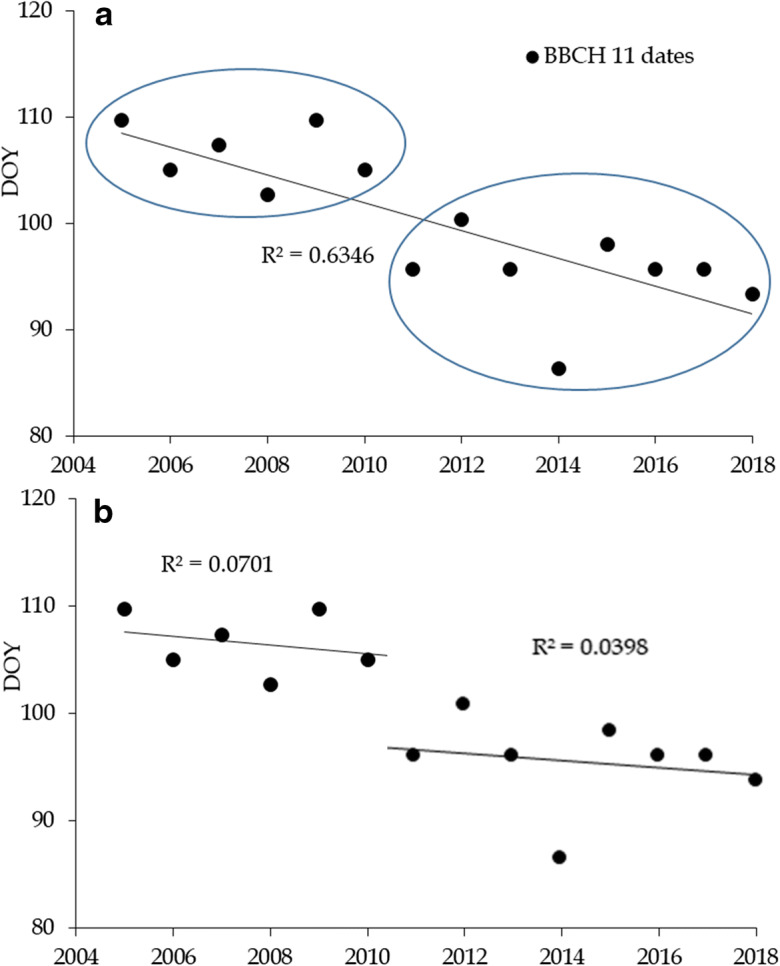
Discontinuity detection in the BBCH11 time series (**a**) in Salix acutifolia and identification of similar regression lines in the two secondary time series (**b**)

Figure 4 a shows an in-depth analysis of the BBCH11 dates during the study period for *S. acutifolia*, which allowed the detection of a marked discontinuity in the BBCH11 time series, identifying 2 sub-periods, BBCH11A, from 2005 to 2010 and BBCH11B from 2011 to 2018, characterized by similar regression lines (Fig. 4b).

## Discussion

Phenological models may produce valuable information for the fields of agriculture and forestry, suggesting better practices and the implementation of appropriate measures to optimize yields (Cola et al. 2014; Rojo and Pérez-Badia 2015). The timing of yearly leaves presence from first leaves unfolded, to shoot growth and fallen leaves, influences the photosynthesis, affecting biomass production, but can also expose plants to the risk of spring late frosts (Polgar and Primack 2011). The leaf development phenomenon is very critical for modelling present and future forest and tree-crop dynamics, since it can determine the availability of food and shelter for many species, creating cascading effects through multiple trophic levels (Wesolowski and Rowinski 2006; Both et al. 2009; Coyle et al. 2010).

The present study allowed the identification of the best phenological models in willow species for which Sigmoid model performed efficiently in simulating spring vegetative phases, BBCH11 and 91 respectively. However, for flowering the performances were not satisfactory probably due to the fact that in deciduous arboreal species with precocious flowering dates at the end of winter-early spring, the reproductive development is particularly influenced by the chilling amounts (flowering induction phenomena) in addition to the successive forcing temperature (after break dormancy). This winter chilling requirement clearly represents an adaptation of the plant in order to prevent the beginning of reproductive development may the frosts during February–March in the Mediterranean area become very dangerous (Larcher 2003).

As regard the calculation of the leaf period (LP) and its subdivision in progressive percentages during the vegetative development, the phenological platform averagely designated the month of June, when full leaf development (BBCH91) was recorded during the study period, as the period that was better simulated by the models. This is probably because this is the phase of the year when the forcing temperatures express their influence mainly on vegetative structures, while precedent phenological phases could be influenced also by winter chilling effects. Successive phenophases such as the senescence ones during September–October could be influenced by summer water availability stress, therefore have a reduced efficiency of model interpretation.

The modelling results showed as the addition of Day Length variable in the interpretative models was significant above all during the senescence phase when the light hours decrease rapidly confirming as photoperiod play an important role in regulating the leaf development of temperate woody plants (Ghelardini et al. 2014). Decreasing day-length induces growth cessation and bud set in many perennial plants (Lagercrantz 2009) and, along with temperature, it may force leaves senescence and shedding (Kozlowski and Pallardy 1997; Tanino et al. 2010; Heide 2011). Several studies indicate that temperature and stress factors may variably interact with photoperiod in controlling the timings of phenological events in woody species, including willow and poplar (Barros and Neill 1987; Molmann et al. 2005; Kalcsits et al. 2009).

As regard the trend analysis results, the BBCH11 time series discontinuity, with the definition of two sub-periods, was due to several positive winter temperature anomalies recorded from 2011 to 2018. In this last 8-year period, only winter 2012 showed a negative temperature anomaly (limited to a polar jet stream phenomenon during 2 weeks of February). All other winters during this period appeared to be averagely milder in the study areas (central Italy), with the winter of 2014 commonly cited as the third warmest winter since 1950.

The capacity demonstrated by the plant to have vegetative growth cessation and critical phenomena, as winter dormancy and differently temperature-mediated, allows to identify possible risk-adverse adaptations to ensure development under variable environmental conditions (Merilä and Hendry 2014; Urban et al. 2016). In temperate plants, the leaf emission phenomenon may also be highly adaptable. In this view, while species with low plasticity and adaptability may suffer under warmer conditions, other species will likely thrive. However, the same phenotypic variation effect is actually discussed mainly in presence of low specific genetic variation, considering that, in the short term, the only way plants can respond to temperatures rising is by adjusting to the new environmental conditions through plasticity, while, in the long term, they achieve this through adaptive evolution, by exploiting both the presence of genetic variation and the seasonal adaptation (Oostra et al. 2018).

*Salix acutifolia* showed phenological development stages more efficiently interpreted through simulation by the utilized statistical models in comparison to *Salix smithiana*, especially during spring (BBCH11 and 91). In this sense, *S. acutifolia* showed highly temperature-mediated phenological traits probably due to a specific phenotypic plasticity that will ensure the survival of the species populations under future climate change projections, even if generalizations are very difficult considering that some species are limited by temperature and photoperiod (Savolainen et al. 2011). In the future climate scenario, the geographical area of distribution of Salicaceae plants is generally likely to shift to high latitudes. Salicaceae is a group of plants with a large ecological amplitude, thanks to which they can adapt to many different ecological environments. However, if global warming becomes more intense, the suitable range of Salicaceae faces the risk of reduction. Species of Salicaceae are, therefore, clearly vulnerable to climate change effects, with contractions in their range being mainly concentrated at low latitudes (Bertrand et al. 2011; Garcia et al. 2013).

Lastly, the phenological derived models will be used to assess the impacts of climate change on the critical development phases in different species, running simulations with the use of climatic data from different forcing scenarios and climate model experiments. Recent simulations of the potential changes in phenological timings may also deliver important information for medium-to-long term planning, suggesting future anticipations of the spring phenophase timings up to about 30 days until the middle of the twenty-first century (Ibanez et al. 2010; Pearson 2019). The growing season length study and forecasting can have important impacts on ecosystem processes, including the uptake of CO_2_, tree growth, microclimate and water movement (White et al. 1999; Morisette et al. 2009). Tree foliage investigations may suggest the ecological consequences of such growing season increase in terms of carbon sequestrations balance due to a contemporary variation of two biological phenomena, photosynthesis and respiration inside forests (Milyukova et al. 2002; Dunn et al. 2007; Piao et al. 2008; Richardson et al. 2010).
